# Ecdysteroids from the Stem Bark of *Vitex doniana* Sweet (Lamiaceae; ex. Verbenaceae): A Geographically Variable African Medicinal Species

**DOI:** 10.3390/antibiotics10080937

**Published:** 2021-08-03

**Authors:** Musa I. Bunu, Derek T. Ndinteh, Jacinta R. Macdonald, Moses K. Langat, Sani M. Isyaka, Nicholas J. Sadgrove, Ingrid Melnikovova, Eloy Fernandez-Cusimamani

**Affiliations:** 1 Department of Applied Chemistry, University of Johannesburg, P.O. Box 17011, Doornfontein, Johannesburg 2028, South Africa; musabunuismaila@gmail.com (M.I.B.); dndinteh@uj.ac.za (D.T.N.); 2 Griffith Institute for Drug Discovery, Griffith University, 46 Don Young Rd., Nathan, QLD 4111, Australia; jacinta.macdonald@griffithuni.edu.au; 3 Royal Botanic Gardens, Kew, Richmond TW9 3DS, UK; m.langat@kew.org; 4 Department of Chemical Sciences, Federal University of Kashere, Gombe State, V2G3+R8V, Barri 771103, Nigeria; isyakamsani@yahoo.com; 5 Department of Crop Sciences and Agroforestry, Faculty of Tropical AgriSciences, Czech University of Life Sciences Prague, Kamýcká 129, 16500 Prague, Czech Republic; melnikovova@ftz.czu.cz

**Keywords:** ecdysteroid, black plum, antimicrobial, malaria, MIC

## Abstract

*Vitex doniana* Sweet is an African medicinal species that is prescribed as an aqueous bark extract to be applied topically or orally to achieve anti-infective outcomes. In select regions it is also taken orally as an antimalarial agent. The aim of the current study was to explore the biological properties of *V. doniana* and isolated compounds in the context of pathogenic bacteria and the protozoan parasite *Plasmodium falciparum*. Three compounds were isolated and assigned by nuclear magnetic resonance spectroscopy as ecdysteroids: (1) 20-hydroxyecdysone, (2) turkesterone, and (3) ajugasterone C. Interestingly, two of these compounds had not previously been identified in *V. doniana*, providing evidence of chemical variability between regions. The bark extract and three ecdysteroids were screened for activity against a panel of pathogenic bacteria associated with skin, stomach and urinary tract infections, and the protozoan parasite *P. falciparum*. The crude extract of the bark inhibited all bacterial strains with MIC values of 125–250 μg.mL^−1^. The three isolated compounds demonstrated less activity with MIC values of 500–1000 μg.mL^−1^. Furthermore, no activity was observed against *P. falciparum* at the screening concentration of 4.8 μg.mL^−1^. Nevertheless, we present a hypothesis for the possible mechanism for symptomatic relief of malarial fever, which may involve reduction of prostaglandin E(1) & E(2) activity in the hypothalamus via modulation of the monoaminergic system. While further studies are required to identify all antimicrobial agents within this plant species and to determine the cytotoxicity of each of these compounds, these data suggest that the traditional application of this species as an antiseptic is valid.

## 1. Introduction

*Vitex doniana* Sweet (Lamiaceae; ex. Verbenaceae) is a highly valued medicinal plant prescribed by herbal healers in sub-Saharan countries of Africa. Numerous tribal communities across Nigeria use the species for ailments that include colic, gastroenteritis, infertility, malaria, diabetes, diarrhea and topical bacterial infections [1,2]. While the active components contributing to these therapeutic applications are unclear, and likely complicated by inconsistencies across the published literature, i.e., one study claims to have characterized saponins and highly polar flavonoids using gas chromatography without derivatization [3], which is impossible, chemical studies performed on members of the genus *Vitex* have identified many ecdysteroids [4] that are believed to play a significant role in these effects [5].

Previous antibacterial studies have demonstrated polar extracts of *Vitex* biota to have antibacterial activity. A water extract from the leaves of a *V. doniana* specimen in Nigeria’s adjoining country, Benin, demonstrated activity against *Staphylococcus aureus* (minimum inhibition concentration (MIC) value of 30 μg.mL^−1^) [6] and a similar outcome was achieved using a butanol extract from stem bark of a Nigerian specimen [7]. Furthermore, a water extract from stem bark of another Nigerian specimen demonstrated activity against Gram-negative species of Enterobacteriaceae (MIC 400 μg.mL^−1^ ) [8]. Oddly, while aqueous leaf extracts demonstrated very low MIC values, an ethanol extract of leaves from yet another Nigerian specimen demonstrated much weaker activity (*S. aureus* MIC value 10 mg.mL^−1^) [9]. While the antimicrobial activity of extracts from the leaves is highly variable across the literature, it remains uncertain if this is due to the extraction solvent or to chemovariation across *V. doniana* biota. However, the current study focuses on the bark, which has less research backing but is regarded as a more important organ in ethnomedicinal use [1].

Some regions of Nigeria and Benin have discovered therapeutic outcomes from *V. doniana* that have not been realized by others [10]. Geographical specificity of medicinal plant prescription is often complicated by secrecy between cultural groups, because it is believed that one group’s intellectual property should not be used freely by other practitioners [11]. However, this assumes that there is uniformity of pharmaceutical potential across the medicinal plant’s geographical range. Different medicinal attributes may be due to location-specific chemistry of the species.

The bark specimen collected for the current study is prescribed by a local healer as a potent anti-infective agent and a treatment for malaria, with the latter being a unique prescription for this species. Given these unique therapeutic recommendations, the objective of the current study was to assess the anti-malarial and antibacterial activity of this biota sample. To answer the question of whether ecdysteroids are related to these therapeutic effects, these compounds were isolated and included in the assays. Furthermore, a chemical comparison to other reports on the same species helps to ascertain if chemical variation is a plausible explanation for geographical specificity of traditional therapeutic prescription, particularly regarding antibacterial and antimalarial use.

## 2. Results and Discussion

Two classes of ecdysteroids are recognized according to the organism from where they are isolated. Ecdysteroids that are isolated from arthropods (insects) are zooecdysteroids; whereas those isolated from plants are phytoecdysteroids. Phytoecdysteroids are present in plants at much higher concentrations than zooecdysteroids are in insects. Nevertheless, both are considered non-toxic to humans [12]. Zooecdysteroids are well-known to regulate arthropod metamorphosis, reproduction, molting and diapause, whereas phytoecdysteroids are speculated to play a role in plant-insect interactions, mainly as phytophagous deterrents [12,13]. However, in mammals, phytoecdysteroids are potent elicitors of anabolic gene expression [12,13].

**Figure 1:**
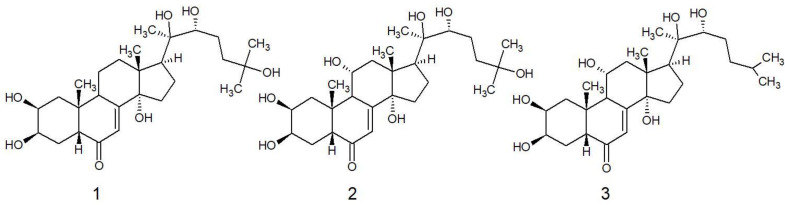
Structures of compounds **1**–**3** isolated from the stem bark of *Vitex doniana*. **1** is 20-hydroxyecdysone, **2** is turkesterone and **3** is ajugasterone C. Ajugasterone C was previously reported in this species.

In the current study the ecdysteroids were isolated from fraction B (see methods). The compounds were identified via their ^13^C NMR spectra (Table 1), which were identical to published values [4] and assigned as the following: (**1**) 20-hydroxyecdysone, (**2**) turkesterone and (**3**) ajugasterone C (Figure 1). Although fully elucidated in previous studies, key features of the NMR spectra are as follows: ^1^H NMR of all three compounds demonstrated five methyl singlets, except for ajugasterone C, which is des-oxidated at position C25 relative to the other two compounds, which gave two methyl doublets 0.70–0.71 ppm. The characteristic olefinic proton at position 7 was shifted downfield to 6.22 in C_5_D_5_N, which is normally further upfield in other solvents (5.81–5.98 ppm).

The ^13^C spectrum includes several shifts in the alcohol region. NMR demonstrated 6 (**1** and **3**) or 7 (**2**) tertiary and quaternary carbon alcohols, particularly the signatory angular hydroxyl carbon at position 14, which is characterised by a relatively downfield shift of 84.0–84.3 ppm for the three structures. The conjugated ketone appeared at >203 ppm and the beta carbon is characteristically drawn downfield to >164 ppm, also on all three structures.

The antimicrobial outcomes derived from the differentially extracted crude methanol fraction against bacteria demonstrated non-discriminate activity between Gram-types, within the margin of 125–250 μg.mL^−1^ (Table 2). These bacterial pathogens are associated with skin, gastrointestinal and urinary tract infections, which are ailments targeted by healers who prescribe the bark extract. In the context of ethnopharmacology, antimicrobial activity of a crude extract in this range is considered moderate to noteworthy [14]. The high polarity of these extracts is an indication that the pharmacokinetic potential (absorption) of the antimicrobial extract is very poor [15], restricting utility to topical and gastrointestinal applications, which is consistent with traditional methods in the context of target anti-infective use.

When 1–3 from the current study were screened against specific bacterial species, MIC values were generally ≥500 μg.mL^−1^, suggesting that they are not the source of the antimicrobial activity demonstrated by the extract. This result is unsurprising because it is already known that ecdysteroids, such as 20-hydroxyecdysone, are not active against microorganisms [16]. However, because acylation augments the antimicrobial activity of ecdysteroids, it should be considered that phase 2 metabolism in humans, leading to acylation of xenobiotic ecdysteroids, may augment systemic antimicrobial effects, contributing to the therapeutic efficacy of the species in traditional use. Because the metabolism of phytoecdysteroids in mammals has not been fully elucidated, the occurrence of an acylation process needs to be confirmed in future studies. However, this does not explain the MIC values achieved for crude extracts. Hence, further testing is required to know if a synergistic effect is responsible, or if other compounds are responsible for this activity. *Vitex doniana* is rich in saponins and authors of antimicrobial studies on the species attribute antibacterial effects to them. Because saponin-enriched extracts have demonstrated non-discriminate activity against Gram-positive [17] and Gram-negative [18] bacteria, then it is likely that saponins are important in these antimicrobial outcomes.

The ecdysteroids (1–3) were also inactive against *Plasmodium falciparum* at 4.8 μg.mL^−1^, a concentration above the usual plasma concentration of these compounds in humans, due to rapid catabolism [12,19]. The high polarity of these compounds slows the rate of absorption from the digestive tract, then they enter plasma for only a brief time, requiring minimal or no conjugation to be catabolized [12] or eliminated via kidneys [15]. One study that focused on ecdysterone confirms that out of the ecdysteroids that survive catabolism, the major xenobiotic eliminated is its unconjugated form, but a significant sub-proportion of the compound is eliminated as metabolized des-oxy deconjugates, including a small amount of 14-deoxy-poststerone, which was tentatively characterized without the side chain [20]. It is unclear if these metabolic des-oxy derivatives could produce significantly different anti-plasmodial outcomes in vivo [5].

Nevertheless, the unique anti-malarial effects of *V. doniana* extracts from the specimen collected in the current study could be attributed to symptom treatment, rather than targeting of the pathogen itself. In this regard, many ‘anti-fever’ species in ethnomedicine confer their effects by anti-inflammatory activity following COX-2 inhibition [21], which reduces prostaglandin E(2) levels in the hypothalamus and attenuates the effects of pyrogens secreted by the invading pathogen [22]. In this regard, the Kenyan study of *V. doniana* [5] demonstrated that the ecdysteroids confer significant in vivo anti-inflammatory effects at 100 mg.kg^−1^ (b.w., PO) in a Sprague Dawley rat model, by oral administration. The specific mechanism of anti-inflammation has not been explored scientifically, but if it is revealed that COX-2 inhibition occurs, then this may help to explain the application to treat malaria. Furthermore, ecdysteroids from *V. doniana* are involved in modulation of the monoaminergic systems of the brain [23]. While this conveys the link to the use of *V. doniana* in the treatment of depression, the monoaminergic system controls thermoregulation and fever modulation as demonstrated in earlier studies via the same pathways (serotonin and dopamine) [24] as those allegedly targeted by the ecdysteroids of *Vitex* [23].

Since the malaria prescription made for the specimen of the current study is not replicated outside of Niger State, it is interesting to know if this could be explained by the incidence of chemical variability. In this regard, a previous study of stem bark from *V. doniana* also isolated ecdysteroids from a specimen growing in the Mau Forest, Kenya [5]. The authors listed seven ecdysteroids, of which only one overlapped with the current study, ajugasterone C. In both studies ajugasterone C was not the major component. The major component expressed by our Nigerian specimen was **1**, which represented an approximate three-fold higher abundance compared to the other ecdysteroids in the extract. Since **1** and **2** were not reported in the *V. doniana* sample taken from Kenya, these data suggest that the composition of ecdysteroids in *V. doniana* differs between these two geographic regions. At this stage it is unclear if this has implications for therapeutic outcomes, because only moderate differences between the ecdysteroids are evident in antimicrobial and anti-inflammatory assays [5]. However, pronounced differences are evident on the monoaminergic response according to the type of ecdysteroid [24], and this could explain regional differences in the use of *V. doniana* in fever treatment.

Furthermore, it is difficult to draw conclusions on relative differences of yield from raw plant material, because the mass of isolates reported in the two studies was not a true reflection of concentration, i.e., not all of the compound can be separated from a mixture and only clean fractions were collected, giving an underestimation of relative abundance in the crude extract. Nevertheless, knowledge of concentration differences between botanical specimens may also be of relevance to the differences in therapeutic claims made by medicinal practitioners.

The anabolic effects of the ecdysteroids may explain the use of the species for such a wide range of ailments. Ecdysteroids are novel anabolic agents that work on the estrogen receptor [25]. They are so effective in human subjects at stimulating the synthesis of muscle fibers that it is recommended that they be listed as category ‘S1 anabolic agents’ in the list of prohibited substances monitored by the World Anti-Doping Agency [19]. Ecdysterone itself also enhances glucose uptake by hepatocytes independent of insulin [26], demonstrating that phytoecdysteroids may be responsible for the anti-diabetic effects described in ethnomedicinal applications of *V. doniana*. Lastly, antimicrobial effects of the crude extract from *V. doniana* and anti-inflammatory effects of the ecdysteroids should be examined as a possible explanation for the symptomatic relief from gastroenteritis, since both inflammation and pathogen infection are associated with this disease.

## 3. Materials and Methods

### 3.1. Plant Material: Collection, Identification and Extraction

The stem bark of *Vitex doniana* was collected in March 2017 from remnant natural vegetation on a private property in the kontagora LGA of Niger state, Nigeria. Identification was made by M. Sabi Idris (Department of Forest Resources Management, Forestry Research Institute of Nigeria), Ibadan Jericho and Yusuf Mukaila (Department of Forestry, Federal College of Wildlife Management, New Bussa, Niger State, Nigeria) where a voucher specimen (Musa/KNT/FHI: 1467) was deposited.

The plant stem bark was air-dried at 37 °C and ground to powder. Solvent extracts were prepared as described by Francis et al., [27] with some small modifications. The three solvents used were hexane, chloroform and methanol. The methanolic extract was obtained differentially after pre-extraction steps. Briefly, 250 g of the powdered stem bark was first extracted in 600 mL of hexane for 72 h, then extracted a second time in 600 mL of chloroform for another 72 h, then finally 600 mL of methanol for 72 h. The filtered methanol extract was concentrated using a rotary evaporator (12.3 g) and stored at 4 °C until used.

### 3.2. Isolation and Characterization of Ecdysteroids

Approximately 150 g of silica gel (60–120 mesh) was manually packed into a column (55 cm × 3 cm) after swelling in hexane. Thereafter, 9.5 g of the methanolic extract was loaded onto a silica gel column in the form of a dry silica mixture (using methanol to combine the two, then evaporated). An initial fractionation process was undertaken using mobile phases successively increasing in polarity, starting with *n*-hexane:ethyl acetate (1:1, fraction A; 1.5 g) then followed by ethyl acetate:methanol (1:1, fraction B; 5.1 g) and finally pure methanol (fraction C; 2.8 g).

Approximately 0.5 g of fraction B was subjected to column chromatography and eluted by a mixture of ethyl acetate and methanol. Chromatography used 100 g of silica, which was swollen in ethyl acetate and packed into a column (55 cm × 3 cm). The sample was loaded then eluted using 400 mL of a mobile phase comprising ethyl acetate (80%) and methanol (20%), followed by 400 mL of mobile phase ethyl acetate (70%) and methanol (30%). Sub-fractions were collected in 10–15 mL volumes then monitored using thin-layer chromatography by comparing the R_f_ value of migrating spots. The TLC plates were stained using Hanessian’s stain (cerium molybdate), and all spots were coloured a blue to black colour. Those fractions with the same R_f_ values were combined. Compounds were eluted in the following sequence: **1** (9.2 mg; fractions 11–15), compound **2** (3.7 mg; fractions 23–29) and **3** (2.5 mg; fractions 16–19) (Figure 1). ^1^H and ^13^C spectra for the isolated compounds were generated on a Bruker 500 MHz NMR spectrometer in *d*-pyridine (C_5_D_5_N, 125 MHz, δ in ppm). Key features of the NMR spectra are as follows: (**1**) 20-hydroxyecdysone, ^1^H; 0.97 (3H s), 1.12 (3H s), 1.27 (3H s, × 2), 1.49 (3H s), 6.20 (1H d, 2.61 Hz), ^13^C spectra in Table 1, (**2**) turkesterone, ^1^H; 1.17 (3H s), 1.22 (3H s), 1.26 (3H s, ×2), 1.49 (3H s), 6.21 (1H d, 2.61), ^13^C spectra in Table 1, (**3**) ajugasterone C, ^1^H; 0.7 (3H d, 6.52 Hz), 0.71 (3H d, 6.52 Hz), 1.19 (3H s), 1.23 (3H s), 1.48 (3H s), 6.22 (1H d, 2.61 Hz), ^13^C spectra in Table 1.

### 3.3. Antimicrobial Assays

The differentially extracted crude methanol-soluble resin was screened against a panel of bacteria (Table 2), following the protocol for the 96-well microtiter plate two-fold serial broth dilution assay, as prescribed by the Clinical and Laboratory Standards Institute [28]. Starting concentrations were 500 μg.mL^−1^, and dimethyl sulfoxide was used to dissolve the resin. Tetracycline was used as a positive control (Sigma Aldrich, Dorset, UK). The ecdysteroid isolates **1–3** were then screened against a smaller selection of bacteria to ascertain if the MIC values of the crude extract were due to these compounds.

### 3.4. Anti-Plasmodial Assays

Anti-plasmodial activity assays were performed in vitro using ^3^H-Hypoxanthine (0.5 μCi/well) incorporation assays and *P. falciparum* 3D7-infected erythrocytes for 48, 72 and 96 h, essentially as previously described [29]. All assays were carried out in 96-well plates, in triplicate at a single concentration of 4.8 µg/mL and were performed on three separate occasions. Positive antimalarial controls chloroquine (Sigma Aldrich, C6628) and clindamycin (Sigma Aldrich, C5269) were included in each assay. Fast action 48 h assays were performed using synchronous parasites at a 1% parasitemia and 1% haematocrit, whereas 72 and 96 h assays were assessed using synchronous parasites at a 0.25% parasitemia, 2.5% final haematocrit or a 0.1% parasitemia, 2% final haematocrit, respectively. After incubation, assay plates were frozen, thawed and harvested onto 1450 MicroBeta filter mats before counting using a 1450 MicroBeta (PerkinElmer). Growth inhibition was determined by comparing the incorporation of test well to that of untreated vehicle controls (0.5% DMSO).

## 4. Conclusions

Chemical variation of the phytoecdysteroids is evident between Kenyan and Nigerian specimens of *V. doniana*, but the potential difference in biological effects described by medical practitioners cannot be explained and remains speculation at this stage. Furthermore, the antimicrobial activity of crude extracts is not explained by the presence of ecdysteroids, but it may involve synergism or a different chemical species altogether. A previously chemical analysis of *V. doniana* describing flavonoids was determined to be spurious, because the authors claimed to have identified saponins by GC-MS. Hence, it is highly likely that the antimicrobial effects are related to the high saponin content of the extracts. Lastly, the ecdysteroids isolated for *V. doniana* in this study demonstrated no anti-plasmodial activity at the starting concentration. However, the mechanism of fever attenuation may be explained by anti-pyrogenic activity, a consequence of the anti-inflammatory effects of ecdysteroids, as demonstrated by others. Ecdysteroids are COX-1 inhibitors, and if enacted in the hypothalamus it has a fever-attenuating effect.

## Figures and Tables

**Table 1:** ^13^C NMR shifts (C_5_D_5_N, 125 MHz, δ in ppm) of compounds **1–3** isolated from the stem bark of *Vitex doniana*, consistent with published values [4]. **1** is 20-hydroxyecdysone, **2** is turkesterone and **3** is ajugasterone C.

Position	1	2	3
1	37.8	39.6	39.6
2	68.0	67.9	68.3
3	67.9	68.2	68.5
4	32.3	32.6	33.0
5	51.2	52.3	52.6
6	203.3	203.7	204.0
7	121.5	122.0	122.4
8	165.9	164.1	164.3
9	34.2	42.5	42.9
10	38.5	39.3	39.9
11	20.9	68.6	68.9
12	31.8	43.9	44.3
13	47.9	47.9	48.3
14	84.0	84.0	84.3
15	31.6	31.7	32.0
16	21.3	21.4	21.6
17	49.9	49.8	49.9
18	17.7	18.7	19.0
19	24.3	24.6	25.0
20	76.6	76.6	76.8
21	21.5	21.4	22.4
22	77.3	77.3	76.8
23	27.3	27.3	30.4
24	42.5	42.4	37.3
25	69.3	69.3	28.3
26	29.8	29.9	23.4
27	29.9	29.8	22.5

**Table 2:** Bacterial and protozoan species, strain and MIC value of the crude methanol extract of *Vitex doniana* stem bark: all values are in μg.mL^−1^, +Cont is tetracycline.

			MIC μg.mL^−1^				
Species	ATCC No.	Gram	Extract	1	2	3	+Cont
*Escherichia coli*	25,922	−	125	>1000	>1000	>1000	0.5
*Klebsiella aerogenes*	13,048	−	125	>1000	>1000	>1000	0.3
*Staphylococcus epidermidis*	12,228	+	125	500	500	500	16.0
*Enterococcus faecalis*	14,506	+	125	500	500	500	32.0
*Proteus mirabilis*	7002	−	250	>1000	>1000	>1000	0.5
*Bacillus subtilis*	19,659	+	250	500	500	500	0.08
*Klebsiella oxytoca*	8724	−	250	500	500	500	1.0
*Enterobacter cloacae*	13,047	+	250	1000	1000	1000	16.0
*Staphylococcus aureus*	25,923	+	250	500	500	500	8.0
*Proteus vulgaris*	33,420	−	125	1000	1000	1000	0.5
*Mycobacterium smegmatis*	14,468	+	250	nt	nt	nt	>32
*Peptostreptococcus asaccharolyticus*	14,963	+	250	nt	nt	nt	>32
*Plasmodium falciparum*	*n*/a	*n*/a	>4.8	>4.8	>4.8	>4.8	*n*/a

## Data Availability

Not applicable.
